# Microglial TLR4-dependent autophagy induces ischemic white matter damage via STAT1/6 pathway: Erratum

**DOI:** 10.7150/thno.49958

**Published:** 2020-07-10

**Authors:** Chuan Qin, Qian Liu, Zi-Wei Hu, Luo-Qi Zhou, Ke Shang, Dale B. Bosco, Long-Jun Wu, Dai-Shi Tian, Wei Wang

**Affiliations:** 1 Department of Neurology, Tongji Hospital, Tongji Medical College, Huazhong University of Science and Technology, Wuhan 430030, China; 2 Department of Neurology, Mayo Clinic, Rochester, MN 55905, USA

In the initially published version of this article 1, the line charts of the 8-arm maze test of the revisiting errors and different arm choice in first 8 entries in Figure 1A, the IF images of the TLR4 KO group in Figure 1C, and the bar chart of CD206 expression in Figure 7B were wrong. The correct Figures are as follows:

The corrections made in this erratum do not affect the original conclusions. The authors apologize for any inconvenience or misunderstanding that this error may have caused.

## Figures and Tables

**Figure 1:**
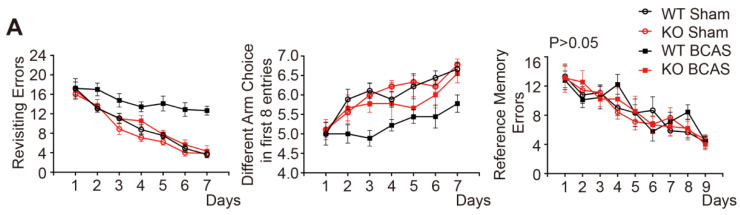

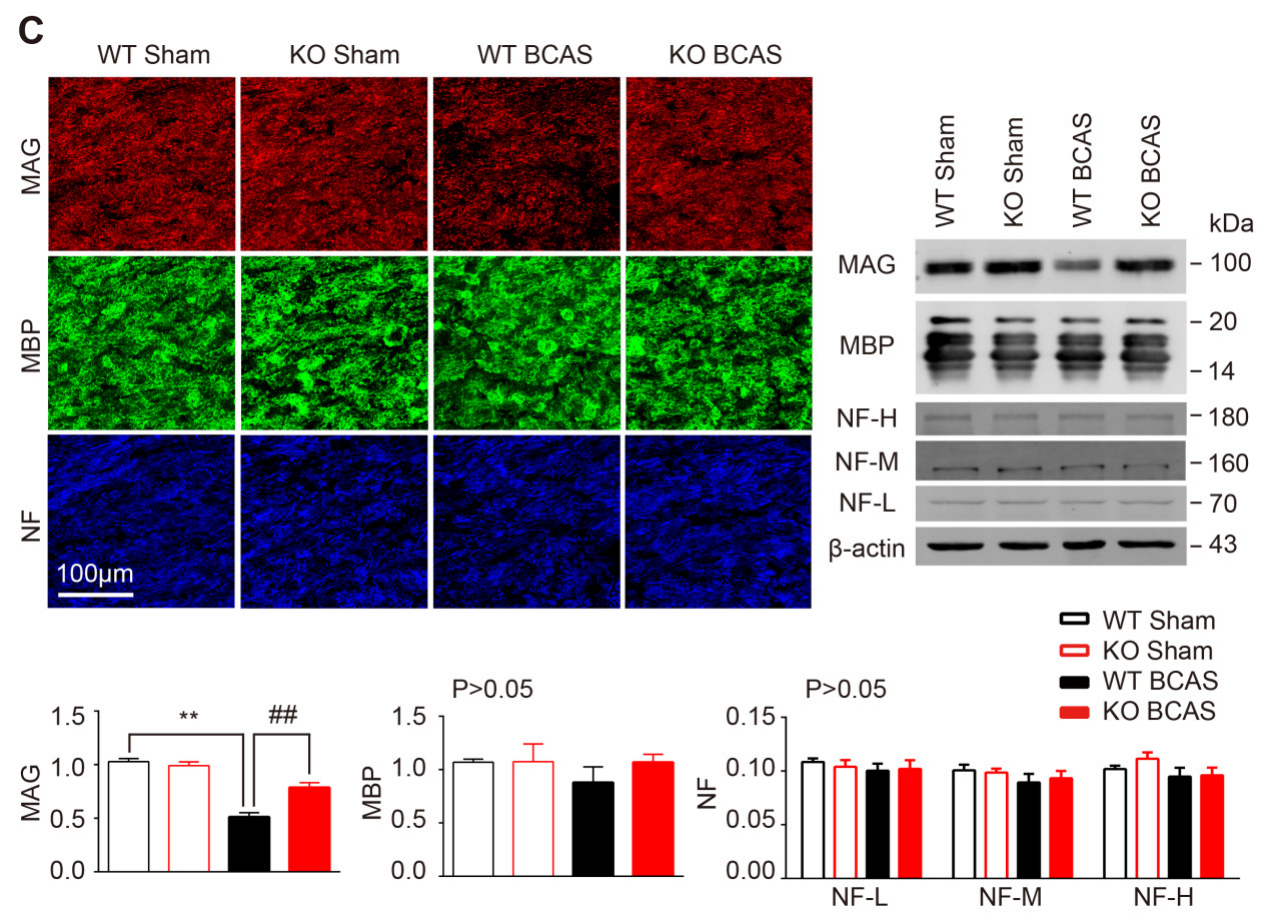
(**A**) The working memory and reference memory of mice were assessed by the 8-arm maze test at 1 month post-injury. WT mice suffered from BCAS made much more revisiting errors (P < 0.001) and less different arm choices (P < 0.001) comparing to the sham-operated mice. TLR4 knockout mice with BCAS made much less revisiting errors (P < 0.001) and more different arm choices (P = 0.007) comparing to the WT group. No impairment in spatial reference memory was revealed between different groups (P > 0.05). Two-way analysis of variance (ANOVA) with repeated analysis, n = 9 per group. (**C**) Representative images depicting immunofluorescent labeling of myelin-associated glycoprotein (MAG), myelin basic protein (MBP), and nerve fiber (NF) in the corpus callosum of coronal slices from different groups. Scale bar, 100 µm. The expression of MAG, MBP and NF (three major NF subunits based upon their molecular mass: the lowest (NF-L), the middle (NF-M) and the highest (NF-H)) was determined by Western blot in mice from different groups. Quantitative analysis of Western blot results was performed. Two-way ANOVA with Dunnett's post-hoc test, **P ˂ 0.01 versus WT Sham, ##P < 0.01 versus WT BCAS. N = 8 per group.

**Figure 7:**
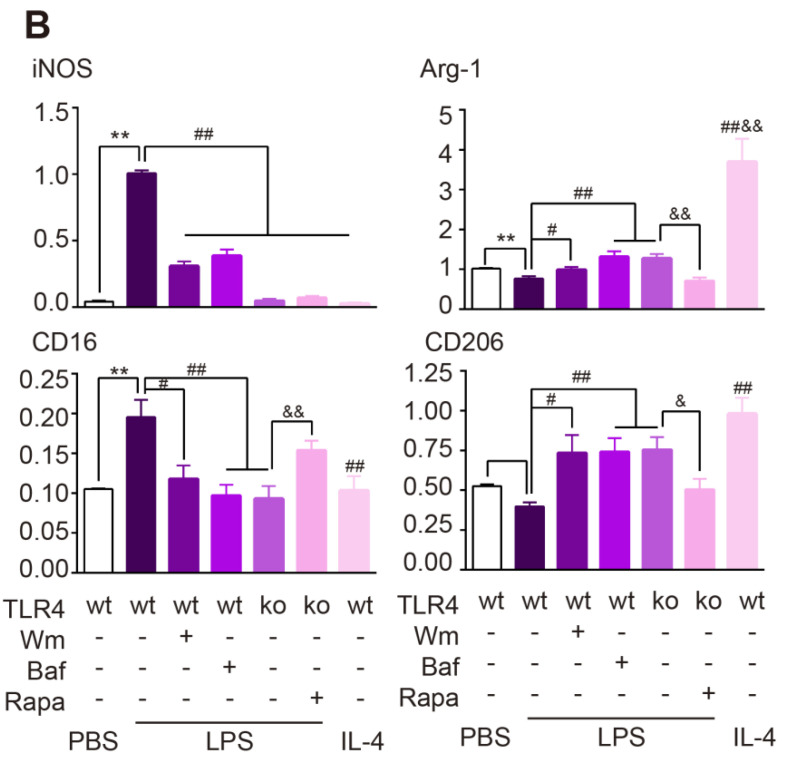
(**B**) Quantitative analysis of Western blot was performed. Two-way ANOVA with Dunnett's post-hoc test, **P ˂ 0.01 versus WT Control, #P ˂ 0.05, ##P ˂ 0.01 versus WT LPS, &P ˂ 0.05 &&P ˂ 0.01 versus KO LPS. N = 8 per group.
